# An updated checklist of *Lysionotus* (Gesneriaceae) and two new species from southwestern China

**DOI:** 10.3897/phytokeys.272.165184

**Published:** 2026-03-23

**Authors:** Jie Huang, Fang Wen, Khang Sinh Nguyen, Chun-Yu Zou, Zhao-Cen Lu, Lei Cai, Wei-Bin Xu

**Affiliations:** 1 Guangxi Key Laboratory of Plant Conservation and Restoration Ecology in Karst Terrain / Nonggang Karst Ecosystem Observation and Research Station of Guangxi, Guangxi Institute of Botany, Guangxi Zhuang Autonomous Region and Chinese Academy of Sciences, Guilin 541006, China Guangxi Institute of Botany, Guangxi Zhuang Autonomous Region and Chinese Academy of Sciences Guilin China; 2 Institute of Ecology and Biological Resources, Vietnam Academy of Science and Technology (VAST), Ha Noi 10072, Vietnam Kunming Institute of Botany, Chinese Academy of Sciences Kunming China; 3 Yunnan Key Laboratory for Integrative Conservation of Plant Species with Extremely Small Populations / Key Laboratory for Plant Diversity and Biogeography of East Asia, Kunming Institute of Botany, Chinese Academy of Sciences, Kunming 650201, China Institute of Ecology and Biological Resources, Vietnam Academy of Science and Technology (VAST) Ha Noi Vietnam

**Keywords:** *Lysionotus guiliangii*, *Lysionotus purpureopunctatus*, plant diversity, taxonomy

## Abstract

By reviewing the taxonomic history of *Lysionotus*, we provide an updated checklist of the species currently accepted in the genus. Currently, 39 species, one subspecies, and four varieties are recorded in the genus. For every species, subspecies, and variety, the scientific name along with the corresponding references is provided. In addition, two new species, *Lysionotus purpureopunctatus* and *Lysionotus guiliangii*, are described and illustrated. *Lysionotus purpureopunctatus* was discovered in Guizhou Province, China, while *L. guiliangii* was found in Yunnan Province, China. *Lysionotus purpureopunctatus* is similar to *L. denticulosus* and *L. oblongifolius*, and *Lysionotus chingii* is morphologically close to *L. gracilis* but can be clearly distinguished by morphological features.

## Introduction

*Lysionotus* D.Don, (1822) (Gesneriaceae) includes around 37 shrubby or suffruticose species, occurring from northern India and Nepal and extending eastwards into Indochina, southern China, and southern Japan. The genus has two main centers of diversity: (1) the karst areas of southern China (Guangxi, Guizhou, and Yunnan) and (2) mountainous regions extending from western Yunnan and southeastern Xizang into northeastern India (Wang 1983; Wei et al. 2010). Our previous study found that *Lysionotus* likely originated in the karst regions of northern Vietnam and southwestern China (diversity center 1) (Huang et al. 2025). Influenced by the East Asian monsoon and past climate fluctuations, *Lysionotus* species later migrated westward and successfully established in the Pan-Himalayan mountain forests (diversity center 2) during the Miocene (Huang et al. 2025).

The known species of the genus *Lysionotus* were divided into three sections, i.e., *Cyathocalyx* W.T.Wang, *Didymocarpoides* W.T.Wang, and *Lysionotus* W.T.Wang (Wang 1983). Since taxonomic revisions by Wang (1983), Hilliard and Burtt (1995), and subsequent updates in “Flora Republicae Popularis Sinicae” (Wang 1990) and “Flora of China” (Wang et al. 1998), the delimitation of the genus and its species has remained largely stable. Since 2000, 11 species in the genus have been newly described, with most of the additional species recently discovered in China and India. China harbors the highest diversity of the genus, with at least 32 species, one subspecies, and four varieties (Tan et al. 2025).

In the previous comprehensive study, we presented a well-resolved phylogeny comprising 27 (about two-thirds) *Lysionotus* species (Huang et al. 2025), revealing three clades in the genus. Among the sequenced taxa, two previously undescribed species, *Lysionotus purpureopunctatus* (as *L. vietnamensis* in Huang et al. 2025) and *Lysionotus guiliangii*, were found. *Lysionotus purpureopunctatus* is morphologically similar to *Lysionotus denticulosus* W.T.Wang and *L. oblongifolius* W.T.Wang in corolla shape, and *Lysionotus guiliangii* is similar to *Lysionotus gracilis* W.W.Sm in corolla shape. After comparing them with these morphologically similar taxa, we confirmed that they represent new species of *Lysionotus*. Thus, we provide comprehensive descriptions and illustrations of these two species here. With the addition of these new species, we present an updated checklist of *Lysionotus*, which now comprises 39 species, one subspecies, and four varieties.

## Materials and methods

The updated checklist was compiled by reviewing more than 20 literature sources concerning the scientific names of all *Lysionotus* species. Following the revision history of the genus, references mainly focus on six comprehensive monographs on *Lysionotus* or the Gesneriaceae family—“Revision of *Lysionotus* in China” (Wang 1983), “*Lysionotus*” (Wang 1990), “Gesneriaceae” (Wang et al. 1998), and “Old World Gesneriaceae. IV. Notes on *Didymocarpus* and *Lysionotus*” (Hilliard and Burtt 1995), “Plants of Gesneriaceae in China” (Li and Wang 2005), “Gesneriaceae of South China” (Wei et al. 2010), and newly described or re-assessed species (e.g., Pal 2000; Lu and Li 2002; Nong et al. 2010; Joe et al. 2017; Taram et al. 2019; Cai et al. 2020; Tian et al. 2020; Akhil et al. 2021; Bui et al. 2022; Chowlu and Krishna 2022; Huang et al. 2022; Chowlu et al. 2023; Lu et al. 2023; Xi et al. 2024; Souvannakhoummane et al. 2025). All scientific names were examined according to the botanical nomenclature for plant taxonomy (Turland et al. 2018), together with online consultation of several online plant name databases, including the Gesneriaceae Resource Centre (https://padme.rbge.org.uk/grc/), World Flora Online (http://www.worldfloraonline.org), The Plant List (http://www.theplantlist.org), Tropicos (https://www.tropicos.org), WFO Plant List (https://wfoplantlist.org/), and the International Plant Names Index (https://www.ipni.org).

We examined herbarium specimens of *Lysionotus* species at CSH, IBK, IBSC, HIB, HITBC, KUN, NAS, and PE (abbreviations follow Thiers, updated continuously; http://sweetgum.nybg.org/science/ih/, accessed on 30 September 2025). Online images of other *Lysionotus* specimens were examined in the Chinese Virtual Herbarium (https://www.cvh.ac.cn/) and JSTOR Global Plants (https://plants.jstor.org). The two new species described here were collected from southwestern China, and voucher specimens were prepared from the type localities or the greenhouse of Guilin Botanical Garden. Voucher specimens of the two new species were preserved in the Herbarium (**IBK**) of Guangxi Institute of Botany and the Herbarium (**KUN**) of Kunming Institute of Botany, **CAS**. Their descriptions draw on examinations of fresh materials, field photographs, and herbarium specimens. Morphological features were described following the standard terminology as defined by Harris and Harris (2001).

A phylogenetic tree of the genus *Lysionotus* (27 sampled species) was reconstructed in our previous study (Huang et al. 2025) based on 649 nuclear gene trees derived from transcriptome data, using ASTRAL v5.6 (Mirarab et al. 2014) to calculate bootstrap support (BS) values for all nodes with 100 replicates. Here, we incorporate the phylogenetic results from that study (details see Huang et al. 2025) to determine the systematic placement of the two new species and to identify their closest relatives.

## Results and discussion

### Classification summary

Following the literature review, the checklist documents 39 species (including the newly described ones), one subspecies, and four varieties belonging to the genus *Lysionotus*. Based on the initial revisions of Wang (1983) and Hilliard and Burtt (1995), 29 (in China) and six species (in India, Nepal, Burma, Thailand, Laos, and Vietnam) of *Lysionotus* were recorded, respectively. After the revisions in “Flora Republicae Popularis Sinicae” (Wang 1990) and “Flora of China” (Wang et al. 1998) and the discovery of additional species and varieties from China to India (see the Methods and the checklist), the checklist of the genus has been updated. Additionally, some species names have been taxonomically revised, e.g., *Lysionotus gamosepalus* W.T.Wang var. *biflorus* A.Joe, Hareesh & M.Sabu has been treated as a synonym of *Lysionotus gamosepalus* W.T.Wang (Lu et al. 2023); *Lysionotus pterocaulis* (C.Y.Wu ex W.T.Wang) H.W.Li was raised to species (Huang et al. 2022); and 15 names have been treated as synonyms of *Lysionotus pauciflorus* Maxim. (see the checklist).

### Phylogenetic placement and morphological comparison for the two new species

**Figure 1.**
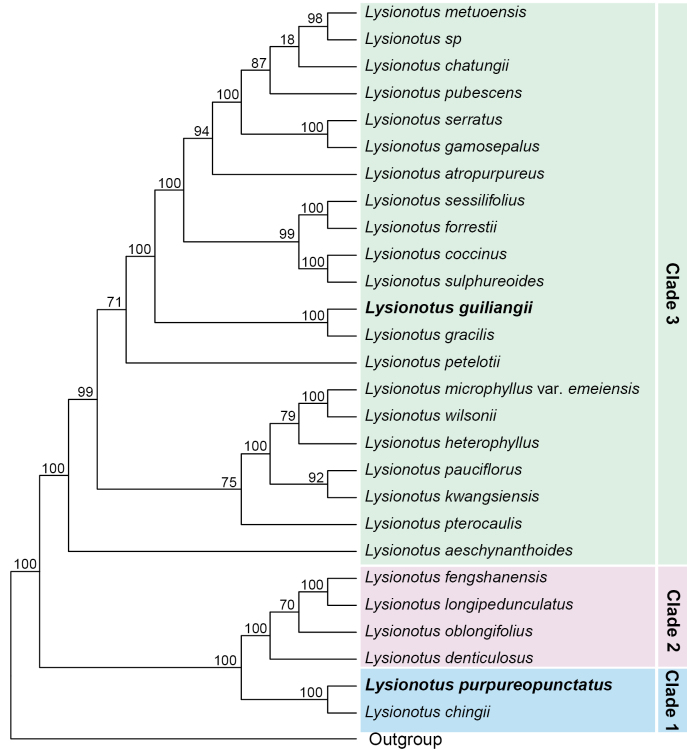
ASTRAL phylogenetic tree of *Lysionotus* based on 649 nuclear genes (modified from Huang et al. 2025). Species names are shown at the tips, with newly described species highlighted in bold. Numbers adjacent to the nodes represent bootstrap support values. Species from different clades are highlighted with colored backgrounds with corresponding clade names shown on the right.

Based on our previously established phylogenetic framework based on 649 nuclear gene sequences of *Lysionotus* (Huang et al. 2025), the two new species, *L. purpureopunctatus* and *L. guiliangii*, are assigned to clade 1 and clade 3, respectively (Fig. 1). The results provided clear and robust support (bootstrap support, BS = 100%) for the generic placement of both new species.

**Figure 2.**
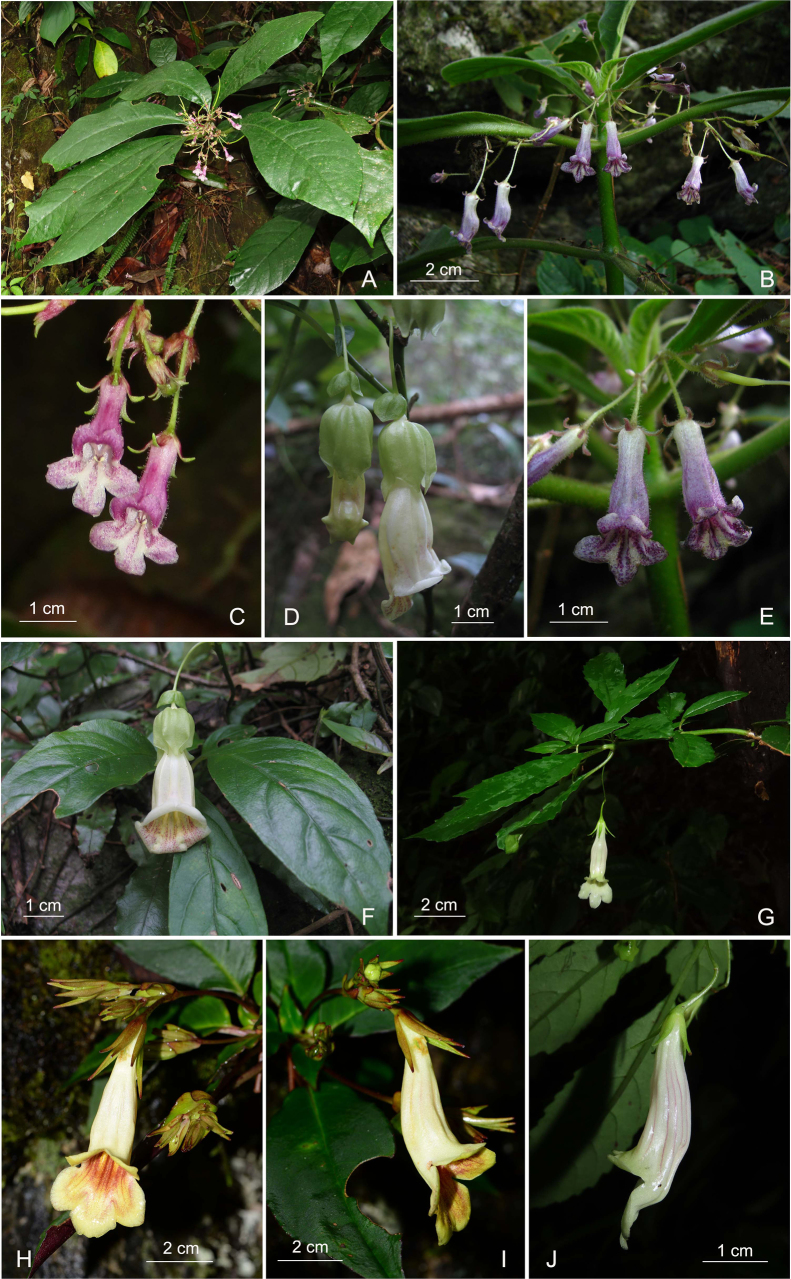
Morphological comparison of five *Lysionotus* species. **A, C**. *Lysionotus oblongifolius*: **A**. Flowering plant; **C**. Front view of flower. **B, E**. *Lysionotus denticulosus*: **B**. Flowering plant; **E**. Front view of flower. **D, F**. *Lysionotus chingii*: **D**. Side view of flower; **F**. Flowering plant with front view of flower. **G, J**. *Lysionotus gracilis*: **G**. Flowering plant with a front view of the flower; **J**. Side view of flower. **H, I**. *Lysionotus petelotii*: **H**. Front view of flower; **I**. Side view of flower. (Photographed by W.B. Xu & X.X. Zhu, and the plate arranged by J. Huang).

Specifically, *L. purpureopunctatus* formed a strongly supported sister relationship with *L. chingii* Chun ex W.T.Wang (BS = 100%) (Fig. 1), which is congruent with its placement in a complete chloroplast genome phylogenetic analysis (Li et al. 2026). However, *Lysionotus purpureopunctatus* typically features a 5-sect calyx from the base and a sparsely glandular-pubescent corolla, very different from the large 5-lobed calyx dissected from above the middle and the glabrous corolla of *L. chingii* (Fig. 2). Morphologically, *Lysionotus purpureopunctatus* is closer to *Lysionotus denticulosus* (Fig. 2) and *L. oblongifolius* (Fig. 2) in floral features but can be distinguished from the latter two in several characters (see diagnosis). Detailed morphological differences between *Lysionotus purpureopunctatus*, *L. chingii*, *L. denticulosus*, and *L. oblongifolius* are shown in Table 1.

**Table 1.** Morphological comparison of *Lysionotus purpureopunctatus*, *L. chingii*, *L. denticulosus*, and *L. oblongifolius*.

Character	*L. purpureopunctatus*	*L. chingii*	*L. denticulosus*	*L. oblongifolius*
Habit	ascending subshrubs, stems 40–65 cm long	prostrate climbing subshrubs or lianas, stems to 9 m long	erect subshrubs, stems 60–130 cm long	erect subshrubs, stems to 80 cm long
Leaf blade	elliptic, 5.6–14.5 × 2.1–6.7 cm, lateral veins 6–10 on each side of midrib	elliptic to narrowly elliptic or oblong, 4.5–13 × 2.2–5 cm, lateral veins 4–6 on each side of midrib	oblong or lanceolate-oblong to ovate, 5.8–18 (25) × 2–6 (9) cm, lateral veins 7–11 on each side of midrib	oblong to obovate-oblong, 9–20 × 2.5–8 cm, lateral veins 7–10 on each side of midrib
Bracts	broadly lanceolate, 3.5–5 × 2–2.5 mm	orbicular-ovate to ovate, 4–7 × 7–9 mm	triangular, ca. 4 × 1.2 mm	narrowly triangular, 2–6 × ca. 0.7 mm
Pedicel	7–14 mm long, sparsely glandular-pubescent	2–7 mm long, glabrous	3–18 mm long, sparsely puberulent	5–15 mm long, puberulent
Calyx	5-sect from base, segments 6–9 × 2.5–4.5 mm, outside pubescent, inside glabrous	5-lobed from above middle, tube 12–18 mm long, lobes 4–5 × ca. 5 mm, glabrous on both sides	5-sect from base, segments 4–5 mm long, outside densely puberulent, inside glabrous	5-sect from base, segments 6–7 mm long, outside rust-brown velutinous, inside glabrous
Corolla	white to greenish, 2.4–3.5 cm long, outside sparsely glandular-pubescent	white or tinged greenish, 4.0–5.5 cm long, outside glabrous	purple-red, ca. 1.7 cm long, outside sparsely puberulent	purple-red, ca. 1.9 cm long, outside puberulent

*Lysionotus guiliangii* was found sister to *L. gracilis* (bootstrap = 100%) in the nuclear gene phylogeny (Fig. 1). But its position was different in the chloroplast genome phylogenetic analysis, in which *L. guiliangii* was recovered as the sister taxon to *L. petelotii* Pellegr. (Fig. 2) (bootstrap = 100%) (Li et al. 2025, 2026). Morphologically, *L. guiliangii* is highly similar to *L. gracilis* in floral features, but the distinguishing features of *L. guiliangii* are its broadly ovate white bracts and densely glandular-pubescent white calyx and corolla, which differ from those of *L. gracilis* (Fig. 2), with linear-oblong green bracts and glabrous calyx and corolla. Detailed morphological differences between *Lysionotus guiliangii*, *L. gracilis*, and *L. petelotii* are shown in Table 2.

**Table 2.** Morphological comparison of *Lysionotus guiliangii*, *L. gracilis*, and *L. petelotii*.

Character	*L. guiliangii*	*L. gracilis*	*L. petelotii*
Leaf blade	elliptic, 10–21 × 5.2–8.1 cm, densely pubescent on both sides	narrowly oblong to lanceolate, 2.2–5.7 × 0.8–1.5 cm, adaxially glabrous, abaxially glabrous, midrib sometimes puberulent	lanceolate to lanceolate-oblong, oblong, or ovate, seldom oblanceolate to obovate, 1.5–9 (11) × 1–2.5 (4.6) cm, glabrous on both sides
Petiole	10–18 mm long	3–11 mm long	4–17 (30) mm long
Bracts	broadly ovate, white, 5–6 × 4–4.5 mm	linear-oblong, greenish, 2–3 × 0.2–0.5 mm	lanceolate, greenish, 3–4 × 0.8–1 mm
Calyx lobes	12–15 mm long, white to greenish outside densely glandular-pubescent	6.0–9.5 mm long, greenish, outside glabrous	11–14 mm long, greenish, outside glabrous
Corolla	white, with purplish stripes, 5.0–5.8 cm long, outside densely glandular-pubescent	white, with purplish stripes, 2.8–3.5 cm long, outside glabrous	purplish to yellow, 5–6.5 cm long, outside glabrous

In summary, based on morphological observations and molecular results, we recognize that the newly collected material represents two undescribed species, and we describe them here as *L. purpureopunctatus* and *L. guiliangii* in *Lysionotus*.

### Taxonomic treatment

#### Lysionotus purpureopunctatus

Taxon classificationPlantaeLamialesGesneriaceae

F.Wen, W.B.Xu & K.S.Nguyen sp. nov.

90A94D3B-8402-5A66-98E1-D8CEAC5FC463

urn:lsid:ipni.org:names:77377903-1

Fig. 3

Lysionotus vietnamensis nom. nud. in Huang et al. 2025.

##### Chinese name.

zǐ bān diào shí jù tái (紫斑吊石苣苔).

**Figure 3.**
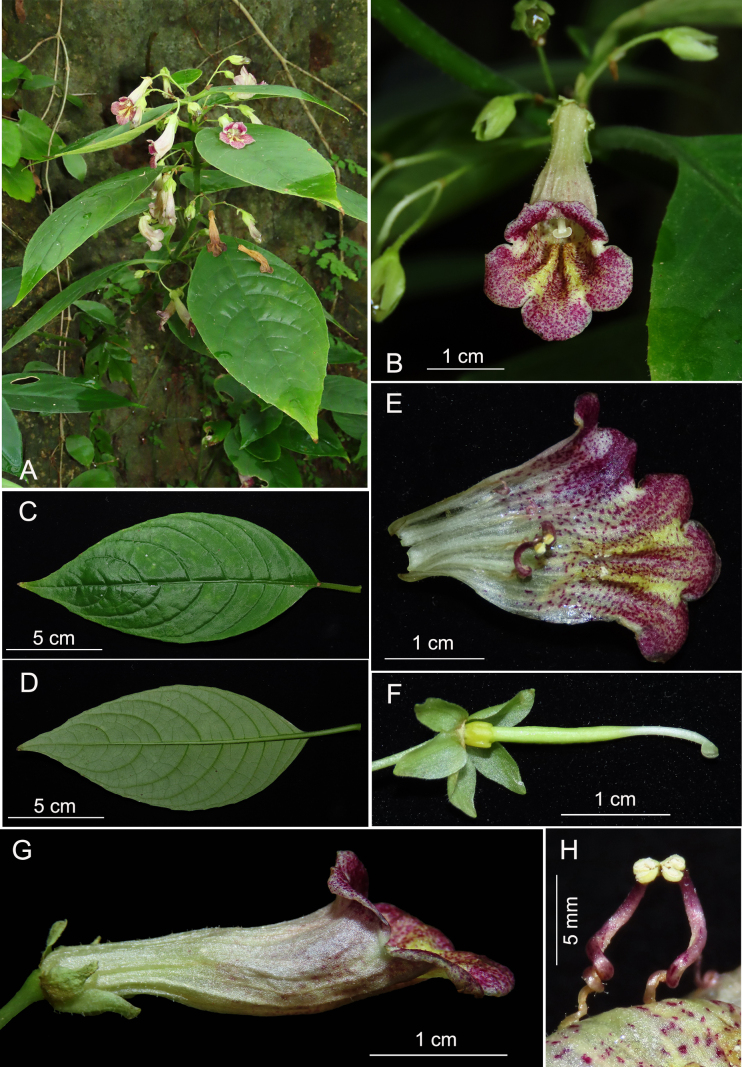
*Lysionotus purpureopunctatus*. **A**. Habit; **B**. Front view of flower; **C**. Leaf surface, adaxial; **D**. Leaf surface, abaxial; **E**. Dissected corolla; **F**. Calyx and pistil; **G**. Side view of flower; **H**. Stamens, showing anthers cohering at the tip. (Photographed by W.B. Xu of cultivated plants at Guilin Botanical Garden, and plate arranged by J. Huang).

##### Type.

China • Guizhou, Libo County, Yaoshan Township, herbarium type specimen prepared from a plant cultivated at Guilin Botanical Garden and introduced from the type locality, 23 October 2020, *W.B. Xu 14229* (holotype: IBK, isotype: IBK).

##### Diagnosis.

*Lysionotus purpureopunctatus* is morphologically similar to *Lysionotus denticulosus* W.T.Wang and *L. oblongifolius* W.T.Wang in corolla shape but can be distinguished from the latter two by being an ascending subshrub (vs. erect), bracts broadly lanceolate (vs. triangular or narrowly triangular); calyx segments ovate to broadly lanceolate (vs. linear-lanceolate); and corolla white to greenish (vs. purple-red).

##### Description.

Ascending subshrubs. Stems 40–65 cm long, 5–8 mm in diam., cylindric, glabrous to very sparsely pubescent. Leaves opposite or whorled in three, petiole 0.6–3.4 cm long, pubescent; leaf blade elliptic, 5.6–14.5 × 2.1–6.7 cm, herbaceous, adaxially glabrous, abaxially pubescent along veins, base cuneate, oblique, margin indistinct serrate, apex acuminate; lateral veins 6–10 on each side of midrib, impressed adaxially, prominent abaxially. Cymes 1–5-flowered; peduncle 0.8–3.5 cm long, sparsely pubescent; bracts 2, broadly lanceolate, 3.5–5 × 2–2.5 mm, margin entire, outside pubescent, inside sparsely pubescent. Pedicel 7–14 mm long, sparsely glandular-pubescent. Calyx 5-sect from base, greenish; segments 6–9 × 2.5–4.5 mm, outside pubescent, inside glabrous. Corolla white to greenish, 2.4–3.5 cm long, outside sparsely glandular-pubescent, inside sparsely puberulent with two yellow ridges; tube tubular, base laterally flattened, 1.4–2.5 × 0.6–0.9 cm; limb distinctly 2-lipped, inside densely purple spotted, adaxial lip 2-lobed to near lobes’ base, lobes broadly ovate, 5–6 × 3–4 mm, abaxial lip 3-lobed, inside purple spotted, middle one lobed near to lobes’ middle, lobes broadly ovate, 6–7 × 4–5 mm. Stamens 2; filaments 9–12 mm long, twisted, purplish, sparsely glandular-pubescent, adnate to ca. 1.1 cm above corolla tube base; anthers ellipsoid, basifixed, coherent at tips, yellowish, thecae 2, nearly parallel, not confluent; connective appendage dotted with purplish glands; staminodes 3, white to purplish, glabrous, lateral ones 6–8 mm long, twisted, adnate to ca. 1.1 cm above corolla tube base; middle one ca. 1.5 mm long, adnate to ca. 1.2 cm above corolla tube base. Disc ringlike, yellowish, margin dentate, 2–3 mm high. Pistil 20–25 mm long, ovary greenish, 14–19 mm long, ca. 1.5 mm across, sparsely glandular-pubescent; style white to greenish, 5–6 mm long, ca. 1 mm across, sparsely glandular-pubescent; stigma depressed globose, ca. 1.5 mm across. Capsule not seen.

##### Distribution and habitat.

*Lysionotus purpureopunctatus* is endemic to southern Guizhou, China, and is currently known only from the type locality and grows on moist rock surfaces in karst evergreen broad-leaved forests at elevations of about 1,000 m.

##### Phenology.

Flowering from October to November.

##### Etymology.

The species epithet refers to the corolla with purple spots inside.

#### Lysionotus guiliangii

Taxon classificationPlantaeLamialesGesneriaceae

Lei Cai, W.B.Xu & J.Huang sp. nov.

419B0F0E-C385-50D4-830D-24F4CF3BFC9A

urn:lsid:ipni.org:names:77377904-1

Fig. 4

##### Chinese name.

guì liáng diào shí jù tái (贵良吊石苣苔).

**Figure 4.**
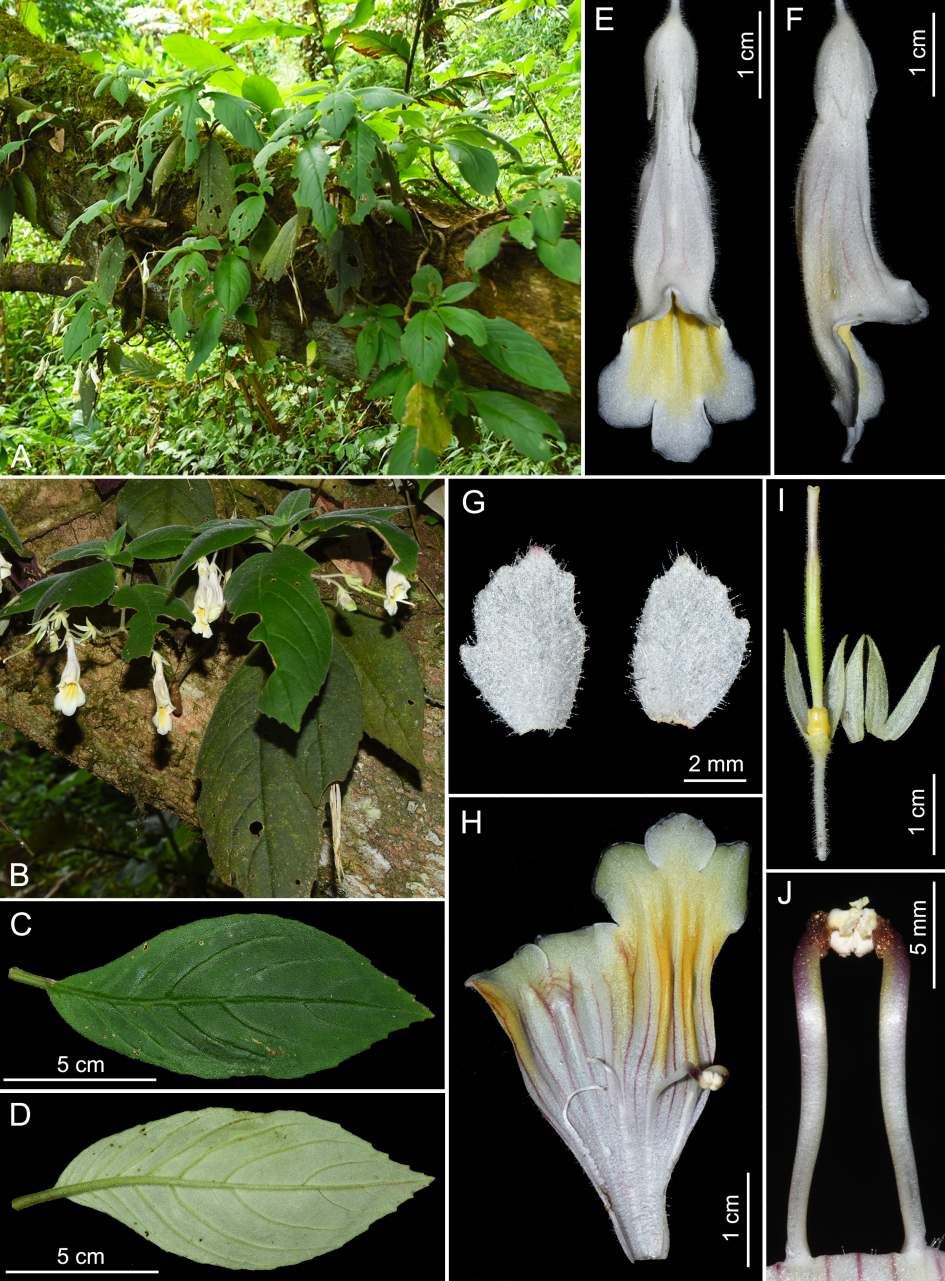
*Lysionotus guiliangii*. **A**. Habitat; **B**. Flowering plant; **C**. Leaf surface, adaxial; **D**. Leaf surface, abaxial; **E**. Front view of flower; **F**. Side view of flower; **G**. Bracts; **H**. Dissected corolla; **I**. Calyx and pistil; **J**. Stamens. (Photographed by L. Cai at the type locality and plate arranged by Z.C. Lu).

##### Type.

China • Yunnan, Pingbian Miao Autonomous County, Yuping Township, near Qigongli, 22°56'57"N, 103°41'12"E, alt. 1, 798 m, 2 September 2020, *L. Cai et al. 2020136* (Holotype IBK, Isotypes IBK and KUN).

##### Diagnosis.

*Lysionotus guiliangii* is morphologically similar to *L. gracilis* W.W.Sm. in corolla shape but can be distinguished from the latter by the larger leaf blade, elliptic, densely pubescent on both sides (vs. narrowly oblong to lanceolate, glabrous on both sides); the bracts, broadly ovate, white (vs. linear-oblong, greenish); the larger corolla, outside densely glandular-pubescent (vs. glabrous).

##### Description.

Epiphytic plants. Stems 25–40 cm long, ca. 1 cm in diam., cylindric, glabrous, apically puberulent. Leaves opposite, petiole 1–1.8 cm long; leaf blade elliptic, 10–21 × 5.2–8.1 cm, herbaceous, densely pubescent on both sides, base cuneate, inequilateral, margin repand to serrate, apex acute; lateral veins 5–8 on each side of midrib, impressed adaxially, prominent abaxially. Cymes 1–7-flowered; peduncle 5–9 cm long, densely pubescent; bracts 2, white, broadly ovate, 5–6 × 4–4.5 mm, margin dentate, densely glandular-pubescent on both sides. Pedicel 10–19 mm long, densely glandular-pubescent. Calyx 5-sect from base, white to greenish; segments 12–15 × 2–3 mm, outside densely glandular-pubescent, inside sparsely glandular-puberulent. Corolla white, 5.0–5.8 cm long, outside densely glandular-pubescent and with purplish stripes, inside sparsely puberulent with two yellow ridges and purplish stripes; tube tubular, base laterally flattened, 3.4–4.0 × 0.8–1.0 cm; limb distinctly 2-lipped, inside yellowish, spreading to white, adaxial lip 2-lobed to near lobes’ base, lobes broadly ovate, 8–10 × 5–6 mm, abaxial lip 3-lobed, inside yellowish, spreading to white, middle one lobed near to lobes’ middle, lobes broadly ovate, 6–8 × 5–6 mm. Stamens 2; filaments 10–13 mm long, white to purplish, glabrous, adnate to ca. 1.5 cm above corolla tube base; anthers ellipsoid, basifixed, coherent at tips, white to yellowish, thecae 2, nearly parallel, not confluent; connective appendage dotted with yellowish to brownish glands; staminodes 3, white, glabrous, lateral ones 8–10 mm long, adnate to ca. 1.5 cm above corolla tube base, apex slightly expanded; middle one ca. 2.0 mm long, adnate to ca. 1.2 cm above corolla tube base. Disc ringlike, yellowish, margin dentate, 2–3 mm high. Pistil 21–25 mm long, ovary white to greenish, 13–16 mm long, ca. 1.5 mm across, densely glandular-pubescent; style white to purplish, 7–8 mm long, ca. 1 mm across, densely glandular-pubescent; stigma hippocrepiform, ca. 1 mm across. Mature capsule and seeds not seen, previously dehiscent valves straight, 7.8–9 cm long.

##### Distribution and habitat.

*Lysionotus guiliangii* is found only in two localities, Pingbian Miao Autonomous County and Hekou Yao Autonomous County, southeastern Yunnan, China, and grows as an epiphyte on moss-covered tree trunks in monsoon evergreen broad-leaved forests at elevations between 1, 700 m and 1, 800 m.

##### Phenology.

Flowering from August to September.

##### Etymology.

The species epithet honors Mr. Gui-Liang Zhang from the Daweishan National Nature Reserve, Hekou, Yunnan, for his contribution to the investigation and conservation of plant diversity in the Daweishan area.

##### Additional specimens examined

**(paratypes)**. China • Yunnan, Hekou Yao Autonomous County, Lianhuatan Township, Baiyan Village, 22°54'57"N, 103°39'50"E, alt. 1, 720 m, 3 September 2020, *L. Cai et al. 2020147* (IBK and KUN).

### *Lysionotus* updated checklist

Compared with previous monographs on *Lysionotus* or the Gesneriaceae family (Wang 1983, 1990; Wang et al. 1998; Li and Wang 2005), we provide an updated checklist for the genus *Lysionotus* arranged alphabetically. For each species, subspecies, and variety in the checklist, the accepted scientific name is provided, along with the original literature and synonyms (≡ denotes homotypic synonyms; = denotes heterotypic synonyms).

1 *Lysionotus aeschynanthoides* W.T.Wang, Guihaia 3(4): 265, 1983.

2 *Lysionotus atropurpureus* Hara, J. Jap. Bot. 48(12): 359, 1973.

3 *Lysionotus calcicola* Phonep., Soulad. & Souvann. Nordic J. Bot. e04620: 4, 2025.

4 *Lysionotus cangyuanensis* C.Liu, W.G.Wang & H.C.Xi, Taiwania 69(4): 445, 2024.

5 *Lysionotus chatungii* Taram, A.P.Das & Tag, Pleione 13(2): 399, 2019.

6 *Lysionotus chingii* Chun ex W.T.Wang, Guihaia 3(4): 279, 1983.

7 *Lysionotus coccinus* G.W.Hu & Q.F.Wang, Nordic J. Bot. 38(11)-e02912: 2, 2020.

8 *Lysionotus confertus* C.B.Clarke, Monogr. Phan. 5(1): 58, 1883.

9 *Lysionotus denticulosus* W.T.Wang, Guihaia 3(4): 264, 1983.

10 *Lysionotus fengshanensis* Yan Liu & D.X.Nong, Nordic J. Bot. 28(6): 720, 2010.

11 *Lysionotus forrestii* W.W.Sm., Notes Roy. Bot. Gard. Edinburgh 10: 185, 1918.

12 *Lysionotus gamosepalus* W.T.Wang, Guihaia 3(4): 278, 1983. = *Lysionotus gamosepalus var. biflorus* A.Joe, Hareesh & M.Sabu, Taiwania 62(4): 337. 2017.

13 *Lysionotus gracilis* W.W.Sm., Notes Roy. Bot. Gard. Edinburgh 10: 186, 1918.

14 *Lysionotus guiliangii* Lei Cai, W.B.Xu & J.Huang (In this study)

15 *Lysionotus hagiangensis* C.H.Nguyen & Aver., Taiwania 67(3): 322, 2022.

16 *Lysionotus heterophyllus* Franch., Bull. Mus. Hist. Nat. (Paris) 5(5): 249, 1899. = *Lysionotus brachycarpus* Rehder, Pl. Wilson. 3(2): 387, 1916.

16-1 *Lysionotus heterophyllus var. lasianthus* W.T.Wang, Guihaia 3(4): 267, 1983. = *Lysionotus pauciflorus var. lasianthus* W.T.Wang, Guihaia 3(4): 276, 1983.

16-2 *Lysionotus heterophyllus var. mollis* W.T.Wang, Acta Phytotax. Sin. 13(2): 69, 1975.

17 *Lysionotus involucratus* Franch., Bull. Mus. Hist. Nat. (Paris) 5(5): 249, 1899.

18 *Lysionotus kingii* (C.B.Clarke) Hilliard, Edinburgh J. Bot. 52(2): 219, 1995. ≡ *Aeschynanthus kingii* C.B.Clarke, Monogr. Phan. 5(1): 31, 1883. ≡ *Trichosporum kingii* (C.B.Clarke) Kuntze in Revis. Gen. Pl. 2: 478, 1891.

19 *Lysionotus kwangsiensis* W.T.Wang, Acta Phytotax. Sin. 13(2): 68, 1975.

20 *Lysionotus levipes* (C.B.Clarke) B.L.Burtt, Edinburgh J. Bot. 52(2): 220, 1995. ≡ *Aeschynanthus levipes* C.B.Clarke, Monogr. Phan. 5(1): 28, 1883. ≡ *Trichosporum levipes* (C.B.Clarke) Kuntze in Revis. Gen. Pl. 2: 478, 1891. = *Lysionotus angustisepalus* W.T.Wang, Guihaia, 3(4): 269, 1983.

21 *Lysionotus longipedunculatus* (W.T.Wang) W.T.Wang, Guihaia 3(4): 261, 1983. ≡ *Chirita longipedunculata* W.T.Wang, Acta Phytotax. Sin. 13(3): 104, 1975.

22 *Lysionotus metuoensis* W.T.Wang, Acta Phytotax. Sin. 17(1): 110, 1979.

22-1 *Lysionotus metuoensis* subsp. *arunachalensis* Chowlu & G.Krishna, J. Jap. Bot. 97(2): 100, 2022.

23 *Lysionotus microphyllus* W.T.Wang, Guihaia 3(4): 270, 1983.

23-1 *Lysionotus microphyllus* var. omeiensis (W.T.Wang) W.T.Wang, Novon 7(4): 429. 1998 ≡ *Lysionotus omeiensis* W.T.Wang, Guihaia 3(4): 271. 1983.

24 *Lysionotus namchoomii* Chowlu, C.H.Nguyen, K.Gogoi & Aver., Turczaninowia 26(3): 161, 2023.

25 *Lysionotus oblongifolius* W.T.Wang, Guihaia 3(4): 263, 1983.

26 *Lysionotus palinensis* G.D.Pal, J. Bombay Nat. Hist. Soc. 97(1): 131, 2000.

27 *Lysionotus pauciflorus* Maxim., Bull. Acad. Imp. Sci. Saint-Pétersbourg, sér. 3, 19(5): 534, 1874. = *Aeschynanthus apicidens* Hance, J. Bot. 21: 167, 1883. = *Trichosporum apicidens* (Hance) Kuntze in Revis. Gen. Pl. 2: 477, 1891. = *Lysionotus carnosus* Hemsl., Gard. Chron., ser. 3 28: 349, 1900. = *Lysionotus cavaleriei* H.Lév., Repert. Spec. Nov. Regni Veg. 6(119–124): 264, 1909. = *Lysionotus warleyensis* E.Willm., Gard. Chron., ser. 3, 54: 125, 1913. = *Lysionotus willmottiae* hort., Gard. Chron., ser. 3, 56: 17, 1914. = *Lysionotus wilsonii* Kraenzl., Repert. Spec. Nov. Regni Veg. 24: 217, 1928. = *Lysionotus hainanensis* Merr. & Chun, Sunyatsenia 2(3–4): 321, 1935. = *Lysionotus pauciflorus var. linearis* Rehder, J. Arnold Arbor. 18(3): 246, 1937. *= Lysionotus ikedae* Hatus., Mem. Fac. Agric. Kagoshima Univ. 7(2): 324, 1970. = *Lysionotus montanus* M.T. Kao, Taiwania 17(2): 158, 1972. = *Lysionotus pauciflorus var. lancifolius* W.T.Wang, Guihaia 3(4): 276, 1983. = *Lysionotus pauciflorus var. latifolius* W.T.Wang, Guihaia 3(4): 275, 1983. = *Lysionotus apicidens* (Hance) T.Yamaz., Fl. Japan 69(2): 115, 1994. = *Lysionotus pauciflorus var. ikedae* (Hatus.) W.T.Wang, Novon 7(4): 430, 1998.

27-1 *Lysionotus pauciflorusvar. indutus* Chun ex W.T.Wang, Guihaia 3(4): 275, 1983

28 *Lysionotus petelotii* Pellegr., Fl. Indo-Chine 4: 503, 1930.

29 *Lysionotus pterocaulis* (C.Y.Wu ex W.T.Wang) H.W.Li, Fl. Yunnan. 5: 553, 1991. ≡ *Lysionotus serratus* var. pterocaulis C.Y.Wu ex W.T.Wang, Guihaia 3(4): 277, 1983.

30 *Lysionotus pubescens* C.B.Clarke, J. Linn. Soc., Bot. 25: 5, 1889. = *Lysionotus wardii* W.W.Sm., Notes Roy. Bot. Gard. Edinburgh 10(49–50): 186, 1918. = *Lysionotus gracilipes* C.E.C.Fisch. Bull. Misc. Inform. Kew 1940(1): 41, 1940.

31 *Lysionotus purpureopunctatus* F.Wen, W.B.Xu & K.S.Nguyen (In this study)

32 *Lysionotus sangzhiensis* W.T.Wang, Guihaia 6(3): 164, 1986.

33 *Lysionotus serratus* D.Don, Edinburgh Philos. J. 7(13): 86, 1822. = *Lysionotus ternifolius* Wall., Pl. Asiat. Rar. 2: 20, 1831. = *Didymocarpus esquirolii* H.Lév., Repert. Spec. Nov. Regni Veg. 9: 328, 1911. = *Hemiboea himalayensis* H.Lév., Repert. Spec. Nov. Regni Veg. 9: 329, 1911. = *Lysionotus himalayensis* (H.Lév.) W.T.Wang & Z.Yu Li., Acta Phytotax. Sin. 30(5): 481, 1992.

34 *Lysionotus sessilifolius* Hand.-Mazz., Anz. Akad. Wiss. Wien, Math.-Naturwiss. Kl. 61: 21, 1925.

35 *Lysionotus sulphureoides* H.W.Li & Yuan X.Lu, Acta Bot. Yunnan. 24(1): 23, 2002.

36 *Lysionotus sulphureus* Hand.-Mazz., Anz. Akad. Wiss. Wien, Math.-Naturwiss. Kl. 61: 20, 1925.

37 *Lysionotus tairukouensis* S.S.Ying, New Taxa New Names 3: 195, 2020.

38 *Lysionotus wilsonii* Rehder, Pl. Wilson. 3(2): 388, 1916.

39 *Lysionotus ziroensis* Nampy, N.Krishna, Amrutha & M.K.Akhil, J. Asia-Pacific Biodivers. 14(1): 119, 2020.

### Insufficiently known species

*Lysionotus aucklandii* H.Low, Sarawak, Inhabitants & Productions: 67, 1848.

It is not recognized because no original publication or specimen can be found, and it has been treated as an unplaced name according to the WFO Plant List.

### Excluded species

*Lysionotus albidus* Blume, Bijdr. Fl. Ned. Ind. 14: 765, 1826. ≡ *Aeschynanthus albidus* (Blume) Steud., Nomencl. Bot., ed. 2, 1: 32, 1840.

*Lysionotus angustifolius* Blume, Bijdr. Fl. Ned. Ind. 14: 765, 1826. ≡ *Aeschynanthus angustifolius* (Blume) Steud., Nomencl. Bot., ed. 2, 1: 32, 1840.

*Lysionotus bijantiae* D.Borah & A.Joe, Taiwania 63(3): 232, 2018. = *Henckelia oblongifolia* (Roxb.) D.J.Middleton & Mich.Möller, Taxon 60(3): 776, 2011.

*Lysionotus griffithii* C.B.Clarke, Monogr. Phan. 5(1): 60, 1883 = *Loxostigma griffithii* (Wight) C.B.Clarke, Monogr. Phan. 5(1): 60. 1883.

*Lysionotus longiflorus* Blume, Bijdr. Fl. Ned. Ind. 14: 766, 1826. ≡ *Aeschynanthus longiflorus* (Blume) A.DC., Prodr. 9: 262, 1845.

*Lysionotus longisepalus* H.W.Li, Bull. Bot. Res., Harbin 3(2): 1, 1983. ≡ *Hemiboeopsis longisepala* (H.W.Li) W.T.Wang, Acta Bot. Yunnan. 6(4): 399, 1984. ≡ *Henckelia longisepala* (H.W.Li) D.J.Middleton & Mich.Möller, Taxon 60(3): 776, 2011.

*Lysionotus mollifolius* W.T.Wang, Guihaia 3(4): 262, 1983. ≡ *Anna mollifolia* (W.T.Wang) W.T.Wang & K.Y.Pan, Fl. Reipubl. Popularis Sin. 69: 487, 1990.

*Lysionotus ophiorrhizoides* Hemsl., J. Linn. Soc., Bot. 26(174): 224, 1890. ≡ *Anna ophiorrhizoides* (Hemsl.) B.L.Burtt & R.A.Davidson, Notes Roy. Bot. Gard. Edinburgh 21: 233, 1955.

*Lysionotus pulchellus* Blume ex C.B.Clarke, Monogr. Phan. 5(1): 34, 1883. = *Aeschynanthus horsfieldii* R.Br., Pl. Jav. Rar. 116, 1840.

## Supplementary Material

XML Treatment for Lysionotus purpureopunctatus

XML Treatment for Lysionotus guiliangii

## Additional information

### Use of AI

No use of AI was reported.

### Author ORCIDs

Jie Huang https://orcid.org/0000-0001-8096-3015

Fang Wen https://orcid.org/0000-0002-3889-8835

Khang Sinh Nguyen https://orcid.org/0000-0001-5171-4140

Chun-Yu Zou https://orcid.org/0000-0001-6004-6551

Zhao-Cen Lu https://orcid.org/0000-0002-1725-0247

Lei Cai https://orcid.org/0000-0002-9251-2745

Wei-Bin Xu https://orcid.org/0000-0002-5602-8753

### Data availability

All of the data that support the findings of this study are available in the main text.
